# Interplay between Alternative Splicing and Alternative Polyadenylation Defines the Expression Outcome of the Plant Unique *OXIDATIVE TOLERANT-6* Gene

**DOI:** 10.1038/s41598-017-02215-z

**Published:** 2017-05-17

**Authors:** Qingshun Q. Li, Zhaoyang Liu, Wenjia Lu, Man Liu

**Affiliations:** 1 Rice Research Institute, Fujian Academy of Agricultural Sciences, Fuzhou, Fujian 350003 China; 2 Key Laboratory of the Ministry of Education for Coastal and Wetland Ecosystems, College of the Environment and Ecology, Xiamen University, Xiamen, Fujian 361102 China; 3 Graduate College of Biomedical Sciences, Western University of Health Sciences, Pomona, CA 91766 USA; 4 Department of Biology, Miami University, Oxford, OH 45056 USA; 5 College of the Environment and Ecology, Xiamen University, Xiamen, Fujian 361102 China; 6 Department of Pediatrics, Dell Medical School, The University of Texas at Austin, Austin, TX 78723 USA

## Abstract

Pre-mRNA alternative splicing and alternative polyadenylation have been implicated to play important roles during eukaryotic gene expression. However, much remains unknown regarding the regulatory mechanisms and the interactions of these two processes in plants. Here we focus on an Arabidopsis gene *OXT6* (*Oxidative Tolerant-6*) that has been demonstrated to encode two proteins through alternative splicing and alternative polyadenylation. Specifically, alternative polyadenylation at Intron-2 of *OXT6* produces a transcript coding for AtCPSF30, an Arabidopsis ortholog of 30 kDa subunit of the Cleavage and Polyadenylation Specificity Factor. On the other hand, alternative splicing of Intron-2 generates a longer transcript encoding a protein named AtC30Y, a polypeptide including most part of AtCPSF30 and a YT521B domain. To investigate the expression outcome of *OXT6* in plants, a set of mutations were constructed to alter the splicing and polyadenylation patterns of *OXT6*. Analysis of transgenic plants bearing these mutations by quantitative RT-PCR revealed a competition relationship between these two processes. Moreover, when both splice sites and poly(A) signals were mutated, polyadenylation became the preferred mode of *OXT6* processing. These results demonstrate the interplay between alternative splicing and alternative polyadenylation, and it is their concerted actions that define a gene’s expression outcome.

## Introduction

Alternative splicing (AS) and alternative polyadenylation (APA) of pre-mRNAs allow organisms to increase their coding potential and regulate gene expression. Recent studies with transcriptome-wide analyses using RNA sequencing have revealed that both AS and APA are highly pervasive in plants: at least 61% of Arabidopsis genes^1^ and about 48% of rice genes^2^ undergo alternative splicing, while 70% of Arabidopsis genes^3^ and around 50% of rice genes^4^ have at least one alternative poly(A) site. Though the mechanisms of AS and APA are less well studied in plants, several conserved *cis*-elements have been identified. Analysis of the 5′ and 3′ splice sites in all known introns of Arabidopsis and rice indicates that these sites are very similar to that of humans, where 5′ splice site contains a conserved GT dinucleotide and 3′ splice site contains a conserved AG dinucleotide^5, 6^. Meanwhile, three classes of poly(A) signals in plant pre-mRNA have been described: the near-upstream element (NUE), the far-upstream element (FUE), and the poly(A) site along with its flanking U-rich element named Cleavage Element (CE)^7^. Growing evidence indicates that splicing and polyadenylation interplay with each other and take place co-transcriptionally^8^. Previous studies have shown that mutation of the polyadenylation signal can impair the *in vitro* splicing of proximal but not the 3′-most exons^9^, while weak 5′ splice site and large intron size can promote the usage of composite exon poly(A) sites^10^. A recent study shows that riboswitch controls the plant gene *THIC* expression by splicing and alternative polyadenylation, indicating a competitive nature between these two processes^11^. All these results demonstrate that splicing and polyadenylation are highly coupled events, however, much remains to be learned regarding the regulatory mechanisms of AS and APA in plants, particularly the interrelationship between these two events.

**Figure 1:**
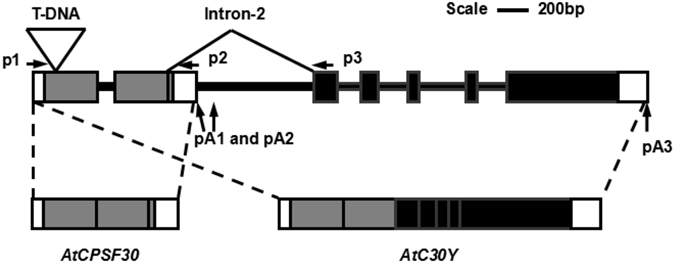
The structure of the *OXT6* gene and RNA transcripts. Exons are indicated in gray or black boxes, UTRs are indicated in white boxes, and introns are indicated in thick lines. The position of the T-DNA insertion in the *oxt6* mutant is indicated above Exon-1. The polyadenylation regions inside Intron-2 (pA1 and pA2) and 3′ UTR (pA3) are indicated with arrows. Positions of primers used for quantitative RT-PCR are labeled above the *OXT6* gene. Primers p1 and p2 are used to detect *AtCPSF30* transcript, while primers p1 and p3 are used to detect *AtC30Y* transcript. This figure is adapted from^14^ with modifications.

The focus of this study is a gene in Arabidopsis named *OXT6* (*Oxidative Tolerant-6*; gene locus ID At1g30460). This gene encodes two mRNAs, which produce two proteins AtCPSF30 and AtC30Y through APA and AS, respectively (Fig. 1). It is a unique phenomenon in plants since its homologue in yeast and animals lacks the AtC30Y homologue coding capacity^12^, thus *OXT6* provides us a good model to investigate the interaction between AS and APA in plants. Intron-2 of *OXT6* contains multiple poly(A) sites. When one of these sites is used, *AtCPSF30* transcript is generated (Fig. 1), which encodes an Arabidopsis ortholog of 30 kDa subunit of the Cleavage and Polyadenylation Specificity Factor (CPSF), serving as a hub of an extensive network of protein-protein interactions in plant mRNA polyadenylation^13^. AtCPSF30 also contains a number of domains for calmodulin binding, nuclease activity, and redox sensing^14^, and may serve as a core to sense some environmental stimuli^15^. On the other hand, if alternative splicing removes Intron-2, a larger transcript *AtC30Y* is produced, which can be translated into a 68 kDa protein containing all but the C-terminal 13 of its 250 amino acids of AtCPSF30 and an additional domain YT521-B (Fig. 1). The function of AtC30Y is less well understood, but the domain YT521-B has been firstly reported as a member in the family of mammalian splicing-associated proteins^16, 17^. The *oxt6* mutant, where a T-DNA insertion in the first exon of the *OXT6* gene disrupts the expression of both transcriptions, displays a reduced sensitivity to oxidative stress^12^, a number of developmental and physiological alterations^18^, and some altered disease responses^19^, suggesting the fact that *OXT6* gene is involved in plant’s survival. Interestingly, a study using high-through-put sequencing methods revealed that over 90% of the poly(A) sites in the *oxt6* mutant were altered^20^, indicating a regulatory role of *OXT6* in plant mRNA alternative polyadenylation. Taken together, these results suggest that *OXT6* can involve in a number of important biological functions such as plant development, hormonal and oxidative stress responses potentially through regulating mRNA 3′ end processing. In this study we show that mutations of the splice sites and/or poly(A) signals that are critical to pre-mRNA processing can significantly change the AS and/or APA patterns of *OXT6*. Through the analysis of a set of mutations, a dynamic interplay among alternative splicing, alternative polyadenylation, and gene expression was revealed.

## Results

### Alternative splicing (AS) and alternative polyadenylation (APA) profiles of the *OXT6* gene

**Figure 2:**
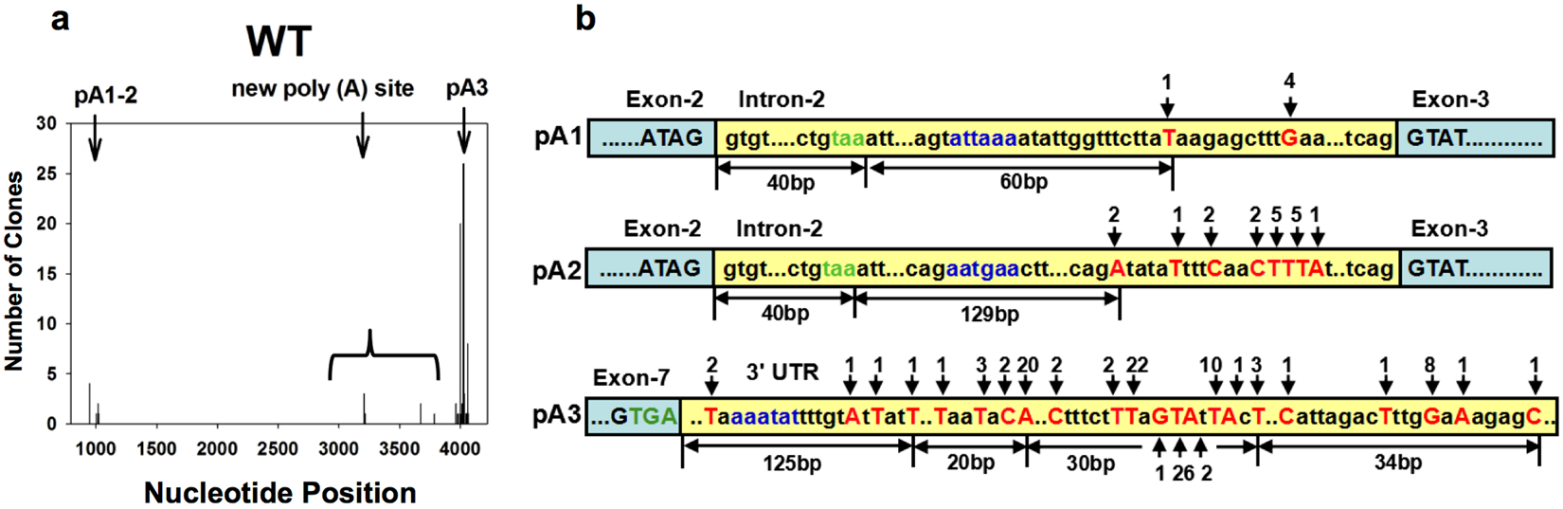
Poly(A) sites and Poly(A) signals of *OXT6* in wild type plants detected by 3′ RACE. (**a**) Poly(A) sites detected by 3′ RACE performed with wild type plants. Nucleotide position of *OXT6* gene (start from ATG) is listed at X-axis, and number of clones polyadenylated at the specific nucleotide is marked by vertical bars at Y-axis. (**b**) Illustrations of poly(A) sites and presumed NUE signals of pA1, pA2 and pA3 in wild type plants. Poly(A) sites [the last nucleotide before the poly(A) tail] are indicated in red; NUEs are indicated in blue; and stop codons are in green. Number of clones sequenced for each poly(A) site is labeled above or below the arrow. Intron-2 and 3′ UTR are shaded in yellow while exons are shaded in blue. Dots (…) represent omitted nucleotide sequences (not drawn in scale).

To define the AS and APA sites, we first confirmed the splice sites and poly(A) signals as well as the AS and APA patterns of *OXT6* gene in wild type plants. Intron-2 of *OXT6* contains typical splice site, where 5′ splice site starts with GT and 3′ splice site starts with AG, as described previously^14^. To detect the poly(A) sites and poly(A) signals in both Intron-2 and 3′ UTR of *OXT6*, 3′ RACE was employed on both *AtCPSF30* and *AtC30Y* transcripts. Two close polyadenylation regions named pA1 and pA2 were detected in Intron-2, and one more (named pA3) was detected in the 3′ UTR of *OXT6* transcripts (Fig. 1 and Fig. 2). Sequencing analysis confirmed that pA1 and pA2 regions were located at approximately from 920 bp to 1050 bp of *OXT6* (start from ATG), containing 10 poly(A) sites (from 23 sequenced transcripts or clones). The pA3 region is located at about from 4000 bp to 4060 bp of *OXT6*, containing 21 poly(A) sites (from 83 clones). All three regions contain presumed NUE signals and groups of poly(A) sites (Fig. 2b). Interestingly, 4 new poly(A) sites (from 7 clones) were detected in Exon-7 of *OXT6* (Fig. 2a), which was not reported before. Taken together, both classical splice sites and poly(A) sites/signals were identified in Intron-2 and 3′ UTR of the *OXT6* transcript for further testing.

### Mutations of splice sites and poly(A) signals change the profiles of AS and APA of *OXT6*

**Figure 3:**
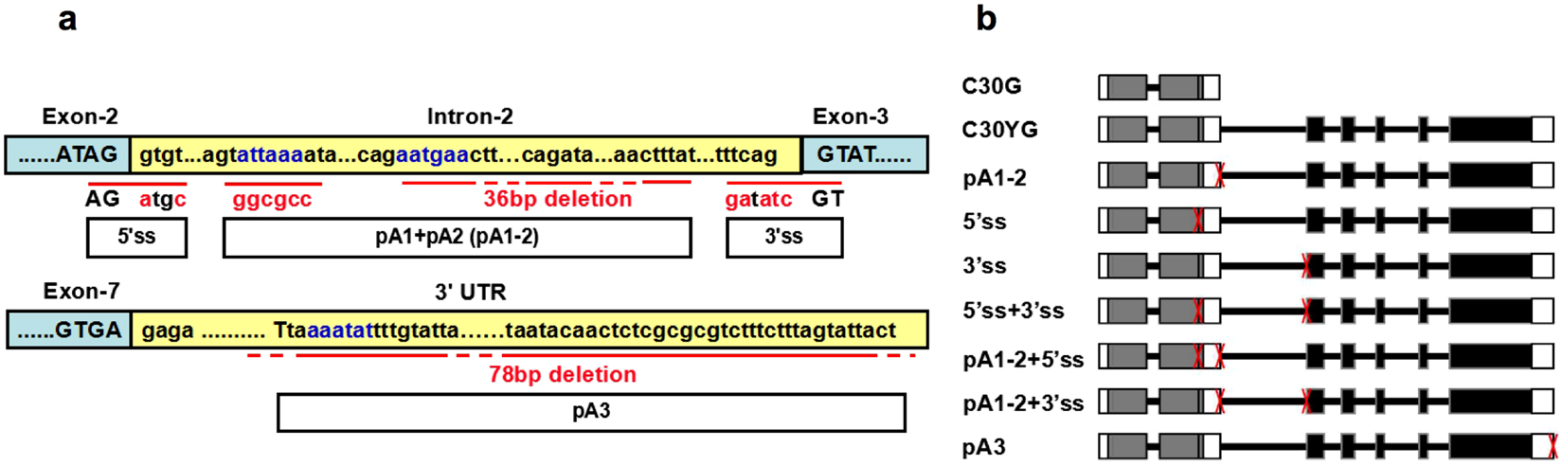
Mutagenesis of the *OXT6* gene. (**a**) Illustrations of the 5′ss, 3′ss, pA1-2 and pA3 mutations. Intron-2 and 3′ UTR are shaded in yellow while exons are shaded in blue. Dots (…) represent omitted nucleotide sequences (not drawn in scale). The presumed NUE are indicated in blue, and the mutated nucleotides are indicated in red. (**b**) Illustrations of the complementary controls and seven *OXT6* mutation constructs. Mutated regions are marked with red “Xs”.

To test the interaction between AS and APA, the full length *OXT6* gene was cloned into a vector, subsequently a set of site-directed mutations were introduced at splice sites and/or poly (A) signals as identified above. The 5′ splice site of Intron-2 was mutated from AG/gtgt to AG/atgc (lower case nucleotides indicate intron sequence, uppercase indicates exon, /indicates splice site), and the 3′ splice site was mutated from tttcag/GT to gatatc/GT (Fig. 3a). The pA1 and pA2 regions were mutated in combination (pA1-2): the presumed pA1 NUE signal were mutated from attaaa to ggcgcc, and a 36 bp of pA2 region was deleted, including the presumed pA2 NUE signal and poly(A) sites (Fig. 3a). A 78 bp of pA3 region, including presumed pA3 NUE signal and poly(A) sites, was also deleted (Fig. 3a). Several mutations were combined to generate double/triple mutations. A total of seven *OXT6* mutation constructs were generated (Fig. 3b) and subcloned into the binary vector pMDC123. The latter was introduced into the *Agrobacterium* strain GV3101 and transformed into the *oxt6* mutant background by the floral dip method to generate transgenic plants (Table 1). In addition, genomic DNA of *AtCPSF30* and *AtC30Y* without any mutation were also introduced into the *oxt6* mutant as complementary controls named as C30G and C30YG, respectively (Fig. 3b and Table 1).

**Table 1:** *OXT6* mutation constructs and transgenic plants used.

Mutant name	Transgenic plants	Mutation target
pA1-2	*oxt6*:: *OXT6*-pA1-2	Poly(A) sites of *AtCPSF30* in Intron-2
5′ss	*oxt6*:: *OXT6*-5′ss	5′ splice site of Intron-2
3′ss	*oxt6*:: *OXT6*-3′ss	3′ splice site of Intron-2
5′ss + 3′ss	*oxt6*:: *OXT6*-5′ss + 3′ss	5′ and 3′ splice sites of Intron-2
pA1-2 + 5′ss	*oxt6*:: *OXT6*-pA1-2 + 5′ss	Poly(A) sites of *AtCPSF30* and 5′ splice site of Intron-2
pA1-2 + 3′ss	*oxt6*:: *OXT6*-pA1-2 + 3′ss	Poly(A) sites of *AtCPSF30* and 3′ splice site of Intron-2
pA3	*oxt6*:: *OXT6*-pA3	Poly(A) site of full-length *AtC30Y*
C30G	*oxt6:: C30G*	No mutation
C30YG	*oxt6:: C30YG*	No mutation

**Figure 4:**
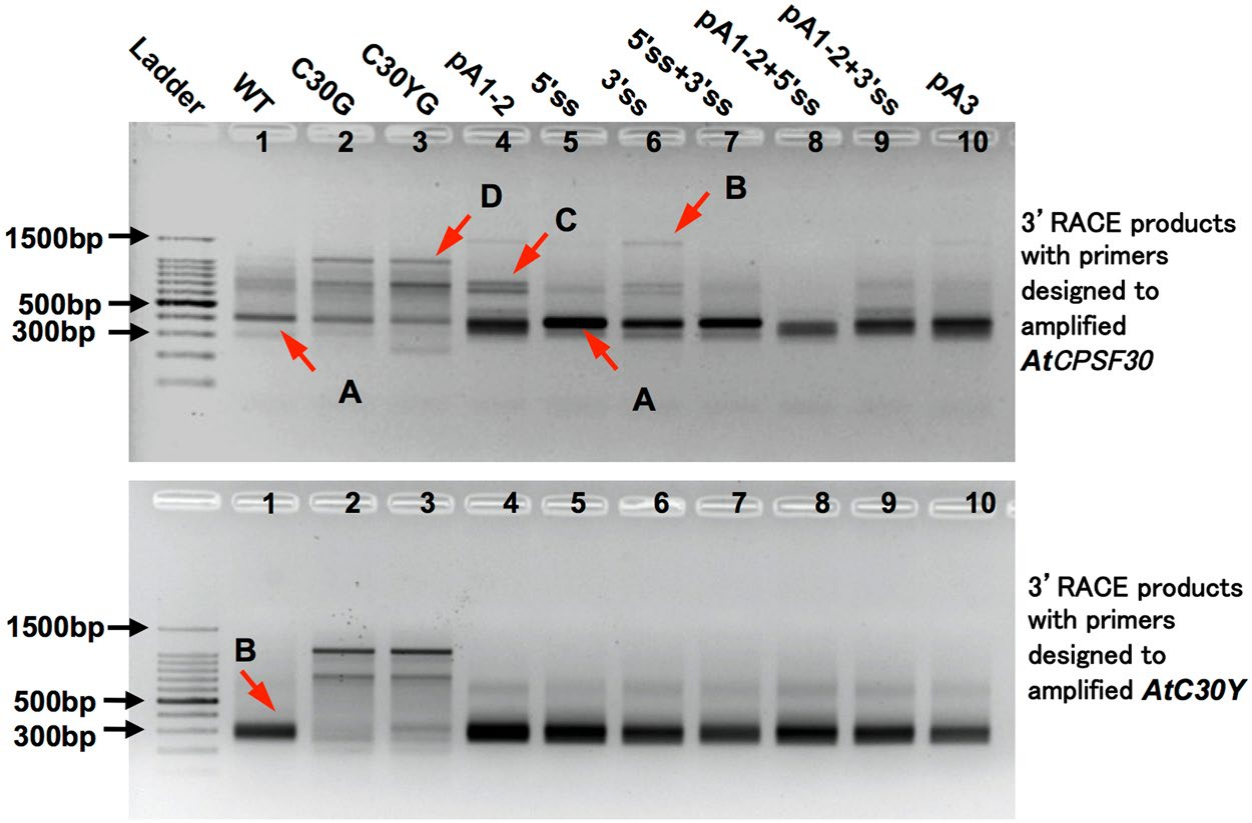
3′ RACE gel image of *OXT6* gene in wild type plant, complementary controls and *OXT6* mutant transgenic lines. Gel pictures of 3′ RACE performed with total RNA isolated from wild type (WT), complementary controls and *OXT6* mutant transgenic lines with primers designed to amplify *AtCPSF30* or *AtC30Y* transcript. Bands marked by A, B, C are transcripts polyadenylated at pA1-2 region, pA3 region, and a new polyadenylation region, respectively. Band marked by D is a nonspecific amplification.

**Figure 5:**
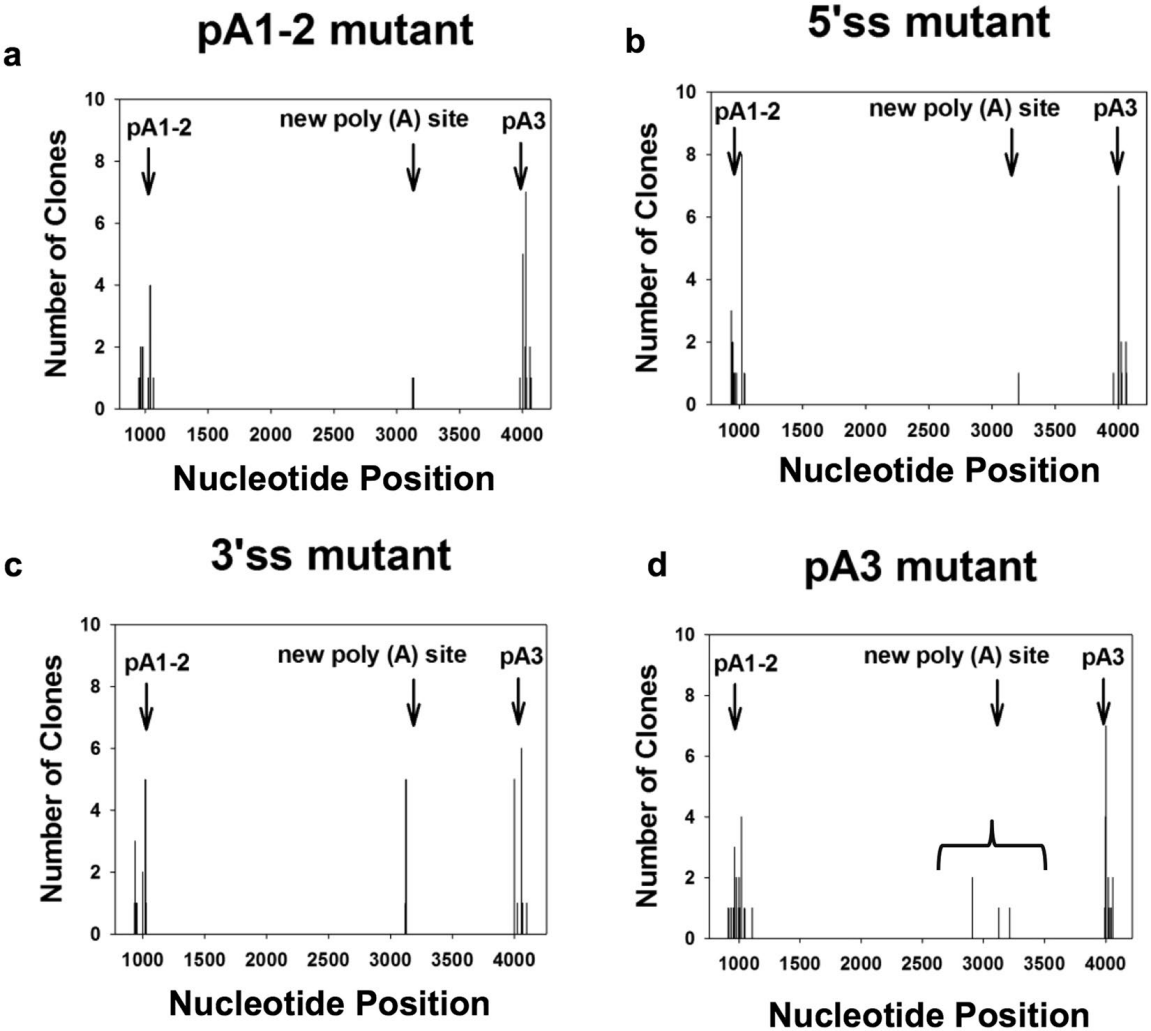
Poly(A) sites detected in *OXT6* mutant transgenic lines. (**a–d**) Poly(A) sites detected by 3′RACE performed with pA1-2, 5′ss, 3′ss and pA3 transgenic plants. Nucleotide position of *OXT6* gene (start from ATG) is listed at X-axis, and number of clones polyadenylated at the specific nucleotide is marked by vertical bars at Y-axis.

Rapid Amplification of cDNA 3′ Ends (3′ RACE) analyses performed on total RNA isolated from pA1-2 transgenic plants revealed an increased fraction of transcripts that are polyadenylated at a new poly(A) region adjacent to the pA1-2 region (Fig. 4, lane 4). This observation was confirmed by sequencing analyses of the 3′ RACE products, where 24 poly(A) sites (44 clones) were detected (Fig. 5a). 11 cryptic poly(A) sites (17 clones) were found in Intron-2 downstream of the previous pA1-2 region, 8 poly(A) sites (20 clones) were detected in pA3 region, and 3 new poly(A) sites (3 clones) were observed in a cryptic poly(A) region in Exon-7 (Fig. 5a). 3′ RACE performed on pA3 transgenic plants revealed 9 cryptic poly(A) sites (19 clones) downstream of the previous pA3 region, 13 ploy (A) sties (20 clones) in the pA1-2 region, and 3 new poly(A) sties (4 clones) in Exon-7 (Fig. 5d). These results indicated that mutations on poly(A) signals can change the poly(A) site selection on both Intron-2 and 3′ UTR of the *OXT6* gene, however in most cases the *AtCPSF30* and the *AtC30Y* transcripts can still be generated using cryptic poly(A) sites. 3′ RACE performed on 5′ss transgenic plants revealed 17 poly(A) sites, 9 of which located in the pA1-2 region (23 clones), 7 in the pA3 region (16 clones), and 1 in Exon-7 (1 clone) (Fig. 5b). Mutation on 3′ splice site results in 10 poly(A) sites in the pA1-2 region (19 clones), 7 in the pA3 region (19 clones), and 2 in Exon-7 (6 clones) (Fig. 5c). These data suggest that mutations on splice sites can also affect the poly(A) site selection on Intron-2.

**Figure 6:**
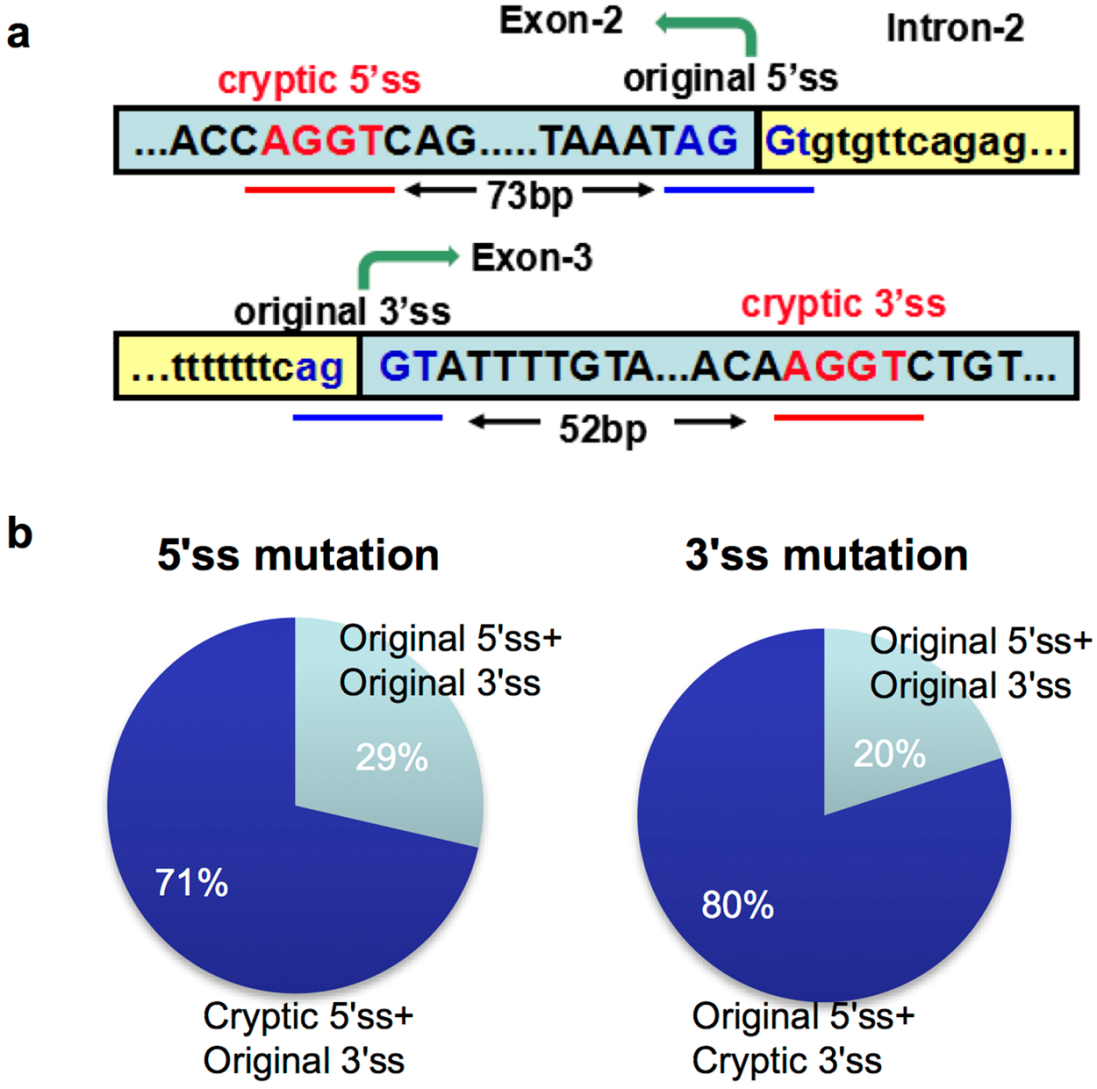
Cryptic splice sites and splicing pattern detected in *oxt6* mutant transgenic lines. (**a**) Cryptic 5′ and 3′ splice sites of Intron-2 in *OXT6* gene detected in transgenic lines with splice site mutations. Original splice sites are indicated in blue while cryptic splice sites are indicated in red. Original exons 2 and 3 are marked by green arrows. (**b**) Splicing pattern detected in transgenic lines with splice site mutations. N = 7 for clones with mutation in 5′ splice site (5′ss). N = 5 for clones with mutation in 3′ splice site (3′ss).

In order to detect the splicing patterns in mutant plants, total RNA of transgenic plants bearing mutations on 3′ splice site and/or 5′ splice site were isolated, reverse transcribed into cDNA, and inserted into vectors for sequencing validation. A cryptic 5′ splice site that was 73 bp upstream of the original 5′ splice site, and a cryptic 3′ splice site that was 52 bp downstream of the original 3′ splice site were identified (Fig. 6a). In transgenic plants with 5′ splice site mutation, 71.4% of 3′ splice site joint with the cryptic 5′ splice site (N = 7) (Fig. 6b), while in transgenic plants with 3′ splice site mutation, 80% of 5′ splice site joint with the cryptic 3′ splice site (N = 5) (Fig. 6b). In both cases, the open reading frame of *OXT6* gene was shifted, though the splicing of the downstream intron (Intron-3) was not affected. These results indicated that mutation on splice sites could alter the splicing pattern of Intron-2; however, Intron-2 can still be removed albeit with altered exon sequences.

### Competitive relationship between AS and APA in Intron-2 of the *OXT6* gene

Since mutations on splice sites and poly(A) signals could change the splicing and polyadenylation patterns of the *OXT6* gene, we then asked if these mutations were interacting with each other. Besides comparing the expression level of the two transcripts, *AtCPSF30* and *AtC30Y*, the relationship between polyadenylation and splicing of Intron-2 could be drawn by comparing the ratio between the two transcripts. We reasoned that if the relationship between polyadenylation and splicing is competition, mutations on polyadenylation signals should promote the splicing process of Intron-2, leading to an increase of the *AtC30Y* transcripts and therefore increase the ratio of *AtC30Y*/*AtCPSF30*. Similarly, mutations on splice sites should stimulate the poly(A) site uses within Intron-2, generating more *AtCPSF30* transcripts and decrease the ratio of *AtC30Y*/*AtCPSF30*. If the relationship between polyadenylation and splicing is cooperation, the result should be opposite.

**Figure 7﻿﻿:**
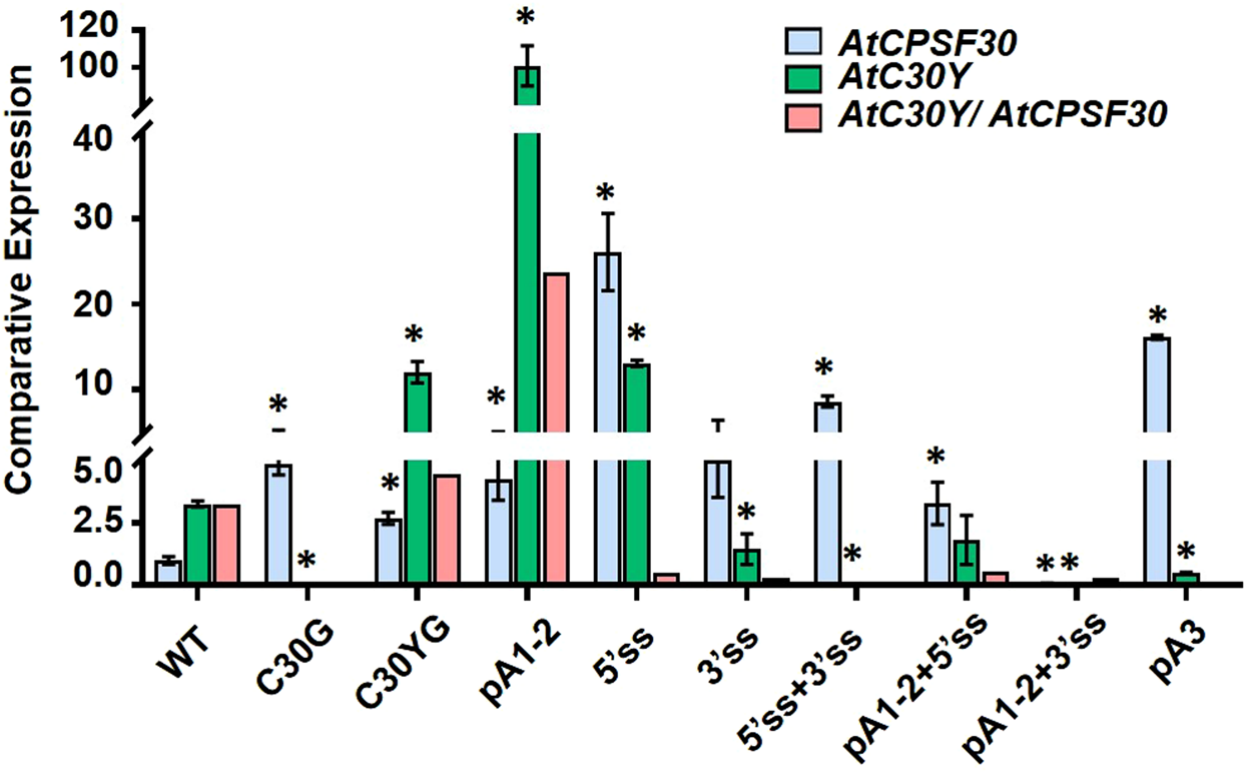
Comparative expression of *AtCPSF30*, *AtC30Y* and the ratio between these two transcripts detected by quantitative RT-PCR. The experiment was performed on at least three biological repeats from T2 generation, and representative results were shown. Bars represent means +/− SD. Two-tail *t*-test results are indicated by (*) with p < 0.05. The significant differences were all compared to the wild type (WT).

To test these possibilities, total RNA was isolated from WT control, C30G and C30YG controls, as well as seven mutant transgenic lines (T2 generation). Expression profiles of *AtCPSF30* and *AtC30Y*, as well as the ratio between these two transcripts were analyzed by quantitative RT-PCR (Fig. 7). Primers specifically designed to detect *AtCPSF30* and *AtC30Y* transcripts even with the usage of cryptic poly(A) sites or cryptic splice sites were labeled in Fig. 1. Gene expression analyses revealed that in the wild type, the expression level of *AtC30Y* is about 3.3 fold more than that of *AtCPSF30*, which is consistent with our previous RNA blotting and protein immunoblot results^14^. Though the expression level of both *AtCPSF30* and *AtC30Y* increased in the C30YG control, the ratio between the two transcripts (which is 4.4) was comparable with that of wild type. No *AtC30Y* transcript was detected in the C30G line as expected. Interestingly, mutations on poly(A) signals in Intron-2 lead to significantly increased *AtC30Y* expression and mildly increased *AtCPSF30* expression, which rose the ratio between *AtC30Y* and *AtCPSF30* to 23.6, indicating a promotion of splicing in the *OXT6* gene processing (Fig. 7). On the contrary, mutations on both 3′ and 5′ splice sites resulted in dramatic increased *AtCPSF30* expression, but the *AtC30Y* transcripts were almost non-detectable, suggesting a drastic switch from splicing to polyadenylation (Fig. 7). These results clearly demonstrated a competitive nature between APA and AS in Intron-2 processing. When one process was suppressed, the other became dominant and thus led to a higher expression level of the corresponding transcript.

Interestingly, mutation on a single splice site resulted in various results. Mutation on 5′ splice site led to increased expression of both *AtC30Y* and *AtCPSF30* transcripts, and the ratio between them was decreased to 0.5 (Fig. 7). This result suggested that both splicing and polyadenylation of Intron-2 were promoted with a weak 5′ splice site, and the processing of *OXT6* gene shifted to the polyadenylation mode. On the other hand, mutation on 3′ splice site resulted in dramatically decreased expression of *AtC30Y* transcripts, but the expression of *AtCPSF30* was not significantly changed (Fig. 7), suggesting that a weak 3′ splice site can significantly impair the splicing of Intron-2, but the process of polyadenylation was not effectively promoted. In conclusion, our data revealed a competitive relationship between APA and AS in Intron-2 processing, and suggested that weakened 5′ splice site could promote both polyadenylation and splicing events while weakened 3′ splice site majorly inhibited Intron-2 removal.

### When both AS and APA signals are weak, APA dominants

Then we asked what happened if the strength of both splice sites and poly(A) signals were weakened. One possibility is that a new balance between AS and APA would be established and the transcripts ratio would be restored to wild type. However, surprisingly, in the mutants of both splice site and polyadenylation signals, a shift from AS to APA was observed (Fig. 7). The ratio between *AtC30Y* and *AtCPSF30* decreased to 0.56 and 0.28, respectively. It is worth noting that the shift from AS to APA was consistent and comparable with that of the single splicing mutations (compare pA1-2 + 5′ss to 5′ss, or pA1-2 + 3′ss to 3′ss in Fig. 7). Particularly, when 3′ splice site was mutated, the splicing process was significantly impaired regardless of the mutation on poly(A) signals. These data suggest that, mutations on splice sites have a more severe effect than that of polyadenylation signals. In another word, when both AS and APA signals are weak, APA becomes a preferred mode.

Finally, mutations in 3′-UTR (pA3) led to inhibited expression of *AtC30Y*, but dramatically increased expression of *AtCPSF30* (Fig. 7), suggesting a competitive relationship between two independent polyadenylation events at Intron-2 and 3′-UTR. Alternatively, due to reduced polyadenylation activity at the 3′-UTR, splicing of Intron-2 was also decreased (potentially via a feedback regulation), promoting polyadenylation at Intron-2.

## Discussion

In our study we found that in the *OXT6* gene, mutations on poly(A) signals in Intron-2 promoted the splicing of the intron while mutations on splice sites stimulated Intron-2 polyadenylation. When both splice site and poly(A) signal were compromised, polyadenylation became the preferred mode. This is the first paper that test the interrelationship between AS and APA in plants, and clearly suggests a competition relationship between these two processes during the *OXT6* gene expression.

The expression outcome of *OXT6* may be determined by several features, including the strength of the splice sites and the poly(A) signals, the presence of potential auxiliary *cis*-elements, as well as the stability of the two transcripts^21^. Intron-2 contains typical splice sites GT-AG, though no highly conserved poly(A) signals (e.g. AAUAAA) were found, some potential signals that were tested to be authentic NUE in other genes were detected. However, it has been reported that a number of *cis*-elements situated in both exons and introns may act as enhancers or silencers in the regulation of alternative mRNA processing^21, 22^. Since we only mutated splice sites and the major poly(A) signal NUE, the ratio between different *OXT6* transcripts might also result from the dynamic interactions between *trans*-acting factors binding to the potential *cis*-elements. Further experimentation is required to reveal such potential signals. In addition, we cannot exclude the possibility that the usage of cryptic poly(A)/splice sites might give rise to transcripts with altered RNA stability. For example, transcripts containing premature stop codon might be eliminated by nonsense-mediated decay. However, according to our data, some transgenic lines such as pA1-2 and 5′ss showed increased expression level of both *AtCPSF30* and *AtC30Y* transcripts (Fig. 7), which should rarely happen if the transcripts were targeted by nonsense-mediated decay. Though the stabilities of these transcripts might be altered by using cryptic poly(A)/splice sites, our results suggested that these changes did not mask the shift between AS and APA modes. To better address this issue, a half-life measurement of different *OXT6* transcripts should be applied in the future.

The interplay between AS and APA may also reflect a self-regulatory expression of the *OXT6* gene. Previous study shows that AtCPSF30, which is generated from APA of the *OXT6* gene, serves as a hub during plant pre-mRNA polyadenylation, around which other subunits assemble into a large complex^15^. Therefore, the APA products of the *OXT6* gene can promote the assembly of the polyadenylation complex, which might in turn stimulate the APA process and increase the expression level of *AtCPSF30*. On the other hand, the YT521-B domain of AtC30Y, which is generated from AS of the *OXT6* gene, has been indicated to associate with splicing^16, 17^. Therefore, the AS product of the *OXT6* gene may potentially involve in the splicing mechanism, and affect the expression level of *AtC30Y* itself, too. This predicated self-regulated mechanism suggests potential different mechanisms of plant mRNA polyadenylation.

In conclusion, our studies for the first time reveal the interrelationship of alternative splicing and alternative polyadenylation in plants. We demonstrated a competition relationship between splicing and polyadenylation during the *OXT6* gene expression, revealed different effects on splice site and poly(A) signal mutation, and suggested a potential distinct role of the *OXT6* gene in plant mRNA 3′ processing.

## Materials and Methods

### Isolation of the *oxt6* mutant

The *oxt6* mutant bearing a transfer-DNA (T-DNA) insertion in the first exon of *OXT6* was identified from a collection of *Arabidopsis thaliana* (ecotype Columbia) from the Arabidopsis Biological Resource Center (Columbus, Ohio, USA) as previously described^12^.

### Plasmid construction and plant transformation

The full length *OXT6* gene (including 2.3 kb of the native promoter) was cloned into Gateway vector pENTR/D-TOPO (Invitrogen Inc.) to generate pENT-C30ASG. Site-directed mutagenesis was performed on the *OXT6* gene with a set of oligonucleotides (Supplementary Table S1) according to the manufacturer’s instructions (QuickChange® II XL Site-Directed Mutagenesis Kit, Stratagene, Inc.). These *OXT6* mutation constructs were converted into the Gateway compatible binary vector pMDC123 using the LR reaction^23^. The binary vectors were then introduced into the GV3101 *Agrobacterium* strain and transformed into *oxt6* mutant background by the floral dip method as previously described^24^. Arabidopsis plants were grown at 24 °C in a 16 hrs light/8 hrs dark cycle. First (T1) and second (T2) generation of transgenic plants were selected on 1/1000 BASTA (ARBICO Organics, Inc.).

### Detection of *OXT6* polyadenylation sites

Rapid Amplification of cDNA 3′ Ends (3′ RACE) was conducted as previously described^3^. Total RNA was extracted from leaves of four-week wild type or T2 transgenic plants and reverse transcribed into cDNA. Two rounds of PCR analyses were performed using one reverse primer [oligo d(T) adaptor] and two forward primers (C30F-1 and C30F-2 for *AtCPSF30*, C30YF-1 and C30YF-2 for *AtC30Y*) (Supplementary Table S1). The second round PCR products were separated by 2% agarose gel electrophoresis. Bands with different sizes were cut and purified by gel extraction kit (Omega) and cloned into pGEM®-T easy vector (Promega) respectively. For each single mutant, more than 40 clones were selected for sequencing and polyadenylation sites analyses.

### Gene expression analyses

Total RNA was extracted from leaves of four-week wild type or T2 transgenic plants and cDNA was synthesized from 1 μg of total RNA using SuperScript-III Reverse Transcriptase (Invitrogen Inc.) and an oligo-d(T) with adapter primer. Quantitative RT-PCR was performed on an iCycler (Bio-Rad Inc.) using SYBR green PCR master mix (Bio-Rad Inc.). The expression of TIP41-LIKE (At4g34270) was used as an internal reference gene^25^. For each transgenic line, at least three independent plants were examined. Primers used are listed in Supplementary Table S1.

## Electronic supplementary material

Supplemental information
