# Supplementary material for: Interplay between Alternative Splicing and Alternative Polyadenylation Defines the Expression Outcome of the Plant Unique OXIDATIVE TOLERANT-6 Gene
Source: Sci Rep. 2017 May 17;7:2052. doi: 10.1038/s41598-017-02215-z (PMC5435732; doi:10.1038/s41598-017-02215-z)
Supplement: Supplementary file 1 — Supplemental information [file 41598_2017_2215_MOESM1_ESM.pdf]

Supplementary Information

Interplay between Alternative Splicing and Alternative Polyadenylation Defines the Expression Outcome of the *OXIDATIVE TOLERANT-6* Gene,

Qingshun Q. Li, Zhaoyang Liu, Wenjia Lu and Man Liu

S1 Table. List of primers used in this study

3' RACE primers

Oligo name	Sequence (5' to 3')
Oligo d(T) with Adaptor	TTCTAGAATTCAGCATTCGCTTCTTTTTTTTTTTTTTTTTV
Oligo d(T) Adaptor	TTCTAGAATTCAGCATTCGCTTC
AtCPSF30 3'RACE-1	CTGGACCTCCACCACCAGTTGA
AtCPSF30 3'RACE-2	CCACAGCTACAAGATAGACCTCA
AtC30Y 3'RACE-1	AGGATGCATCACATGACATGGA
AtC30Y 3'RACE-2	GAAGAGAGTGAAAGTGAAGAC

Mutagenesis primers

Oligo name	Sequence (5' to 3')
OXT6 pA1	GTTGTTATTGGTTCAGTGGCGCCATATTGGTTTCTTATA
OXT6 pA1-comp	GCTCTTATAAGAAACCAATATGGCACTGAACCA
OXT6 pA2	GAATGTAACTGGTTATTTGCAAGTTCAGTATCTACCTGATTTTAGGATAATTTTTC
OXT6 pA2-comp	GAAAAATTATCCTAAAATCAGGTAGATACTGAACTTGCAAATAACCAGTTACATTC
OXT6 5'ss	GCCTCAAGGGGTAAATAGATGCGTTCAGAGTCCTAAGGT

OXT6 5'ss-comp	ACCTTAGGACTCTGAACGCATCTATTTACCCCTTGAGGC
OXT6 3'ss	CTCTTCATAGGTAATCCCTTACATAATTTAGGATCTTGTTTTGATATCGTATTTTGTAGTTAAAAGTAACAATCGAG
OXT6 3'ss-comp	CTCGATTGTTACTTTTAACTACAAAATACGATATCAAAACAAGATCCTAAATTATGTAAGGGATTACCTATGAAGAG
OXT6 pA3	AGCGGCAGGTTGTTGGTGGTCTATGGCATTAG
OXT6 pA3-comp	CTAATGCCATAGACCACCAACAACCTGCCGCT

Real-time RT-PCR primers

Oligo name	Sequence (5’ to 3’)
Tlp41-F (Internal Reference)	GTGAAAACTGTTGGAGAGAAGCAA
Tlp41-R (Internal Reference)	TCAACTGGATACCCTTTCGCA
AtCPSF30 and *AtC30Y*-qPCR-F	CCGCCTGAAAACTCTTCCT
AtCPSF30-qPCR-R	TGAACCAATAACAACGTCTTGA
AtC30Y-qPCR-R	AGCTTCATTGCTCCTTTGTG
